# The Effect of Orally Administered Multi-Strain Probiotic Formulation (*Lactobacillus*, *Bifidobacterium*) on the Phagocytic Activity and Oxidative Metabolism of Peripheral Blood Granulocytes and Monocytes in Lambs

**DOI:** 10.3390/ijms25105068

**Published:** 2024-05-07

**Authors:** Roman Wójcik, Joanna Małaczewska, Dawid Tobolski, Jan Miciński, Edyta Kaczorek-Łukowska, Grzegorz Zwierzchowski

**Affiliations:** 1 Department of Microbiology and Clinical Immunology, Faculty of Veterinary Medicine, University of Warmia and Mazury in Olsztyn, Oczapowskiego 13, 10-718 Olsztyn, Poland; brandy@uwm.edu.pl (R.W.); joanna.malaczewska@uwm.edu.pl (J.M.); edyta.kaczorek@uwm.edu.pl (E.K.-Ł.); 2 Department of Internal Diseases with Clinic, Faculty of Veterinary Medicine, University of Warmia and Mazury in Olsztyn, Oczapowskiego 14, 10-957 Olsztyn, Poland; dawid.tobolski@uwm.edu.pl; 3 Department of Sheep and Goat Breeding, Faculty of Animal Bioengineering, University of Warmia and Mazury in Olsztyn, Oczapowskiego 5, 10-917 Olsztyn, Poland; micinsk@uwm.edu.pl; 4 Department of Biochemistry, Faculty of Biology and Biotechnology, University of Warmia and Mazury in Olsztyn, Oczapowskiego 1A, 10-719 Olsztyn, Poland

**Keywords:** lambs, phagocytosis, oxidative burst, granulocytes, monocytes, probiotic

## Abstract

Probiotic feed additives have attracted considerable research interest in recent years because the effectiveness of probiotics can differ across microbial strains and the supplemented macroorganisms. The present study was conducted on 16 lambs divided equally into two groups (C—control and E—experimental). The examined lambs were aged 11 days at the beginning of the experiment and 40 days at the end of the experiment. The diet of group E lambs was supplemented with a multi-strain probiotic formulation (*Lactobacillus plantarum* AMT14, *Lactobacillus plantarum* AMT4, *Lactobacillus rhamnosus* AMT15, and *Bifidobacterium animalis* AMT30), whereas group C lambs did not receive the probiotic additive. At the beginning of the experiment (day 0) and on experimental days 15 and 30, blood was sampled from the jugular vein to determine and compare: phagocytic activity (Phagotest) and oxidative metabolism (Phagoburst) of peripheral blood granulocytes and monocytes by flow cytometry. An analysis of the phagocytic activity of granulocytes and monocytes revealed significantly higher levels of phagocytic activity (expressed as the percentage of phagocytic cells and mean fluorescence intensity) in lambs that were administered the multi-strain probiotic formulation compared with lambs in the control group. The probiotic feed additive also exerted a positive effect on the oxidative metabolism of both granulocytes and monocytes (expressed as the percentage of oxidative metabolism and mean fluorescence intensity) after stimulation with *Escherichia coli* bacteria and with PMA (4-phorbol-12-β-myristate-13-acetate). These findings suggest that the tested probiotic formulation may have a positive effect on the immune status of lambs.

## 1. Introduction

Antibiotics have increased life expectancy, improved the quality of human life, and significantly contributed to animal health and livestock production efficiency in recent decades. Despite these advantages, the widespread use of antibiotics has led to growing levels of antimicrobial resistance (AMR), in particular, to first-line antibiotics [1]. Antimicrobial resistance poses a significant global problem in both human and veterinary medicine [2]. To address this issue, the use of subtherapeutic doses of antibiotics as growth promoters in animal nutrition was already banned by the European Union (EU) in 2003 (Regulation (EC) No. 1831/2003 of the European Parliament and of the Council of 22 September 2003 on additives for use in animal nutrition) [3]. Despite the advantages of restricted antibiotic use in animal production, antimicrobials are still used in the treatment of many infectious diseases in humans and animals. 

Numerous efforts are being made to develop alternative treatments that could effectively replace antibiotics used for growth promotion and disease prevention, thus protecting human and animal health and minimizing the risk of infections [4,5]. Numerous protocols and methods have been proposed to eliminate pathogenic microorganisms, including immunotherapeutic methods (using monoclonal antibodies that target specific pathogens and toxins without affecting the entire microbiome) [6], immunomodulation (to stimulate the host immune response) [7,8,9,10,11], and vaccines (vaccination programmes can significantly reduce the prevalence of infectious diseases in human and animal populations) [6]. In addition, alternative antimicrobial treatments (such as antimicrobial peptides, silver nanoparticles, and other unconventional compounds) have been shown [12] to be an effective alternative to antibiotics in medicine and agriculture.

On the other hand, more sophisticated approaches have gained more attention from researchers, including the use of bacteriophages (viruses that selectively target and eliminate bacteria) [13], probiotic bacteria alone or in combination with various additives in the form of prebiotics, symbiotics, and postbiotics [14].

Probiotic preparations can be easily administered to animals as feed additives, and they continue to attract growing interest in the livestock industry. Probiotics are beneficial microorganisms (mostly bacteria) that have been divided into three main groups based on their type and origin. Lactic acid bacteria (LAB), including *Lactobacillus* spp. (such as *L. acidophilus*, *L. casei*, and *L. rhamnosus*), *Bifidobacterium* spp. (such as *B. bifidum*, *B. breve*, and *B. longum*), and *Streptococcus* spp. (such as *S. thermophilus*) constitute the largest group of probiotics [15]. The second group consists of *Saccharomyces boulardii* [16], and the third group comprises other microorganisms, including *Bacillus coagulans*, *Escherichia coli* Nissle 1917 (Mutaflor), *Enterococcus faecium*, and *Propionibacterium freudenreichii* [17].

Probiotics deliver numerous benefits for the host organism. Their earliest recognized benefits include increased resistance to infections, stimulation of the immune system, and nutrient synthesis [18], whereas the anticarcinogenic [19], antioxidant [20], anti-inflammatory [21], modulatory [22], and antibacterial [23] effects of probiotics have been described only recently. Preliminary research has shown that probiotics may possess antimicrobial activity, but their clinical efficacy in treating infections has not been extensively studied [24]. However, there is evidence to indicate that probiotics can be useful in the prevention and treatment of infectious diseases [25]. 

Probiotics exert numerous effects on the gastrointestinal tract (GIT) and gut-associated lymphoid tissue (GALT), which modulate intestinal function and immune responses by enhancing activation, adjustment, or tolerance [26,27]. Probiotics produce many bactericidal compounds that eliminate pathogens, adhere to the intestinal epithelium, and interact with pathogens and their toxins. Probiotics enhance the viability of epithelial cells, and improve barrier defences and the immune response of the intestinal epithelium, thus promoting the homeostasis of gastrointestinal mucosa [28]. 

However, the effectiveness of probiotics can vary as probiotic strains may differ in their ability to stimulate immune processes and therefore offer different health benefits. Importantly, the effects of bacterial strains on selected phases of phagocytosis in lambs have never been studied in the literature. *Lactobacillus* and *Bifidobacterium* strains are undoubtedly the most widely used dietary supplements and additives in human and animal diets worldwide [15]. These bacteria provide numerous health benefits by exerting antibacterial, antiviral, and antifungal effects and by reducing the ability of pathogens to bind to host receptors. However, the regulation of the immune response appears to be the most important mechanism by which these probiotics influence the host organism.

Thus, the aim of this study was to evaluate the effect of a multi-strain probiotic formulation containing three *Lactobacillus* strains (*Lactobacillus plantarum* AMT14, *Lactobacillus plantarum* AMT4, and *Lactobacillus rhamnosus* AMT15) and the *Bifidobacterium animalis* AMT30 strain on selected parameters of cellular innate immunity (phagocytosis and oxidative burst of peripheral blood granulocytes and monocytes) in lambs.

## 2. Results

### 2.1. Quantitative Determination of Granulocytes’ Uptake of Bacteria (Phagocytosis) in Lambs

**Figure 1:**
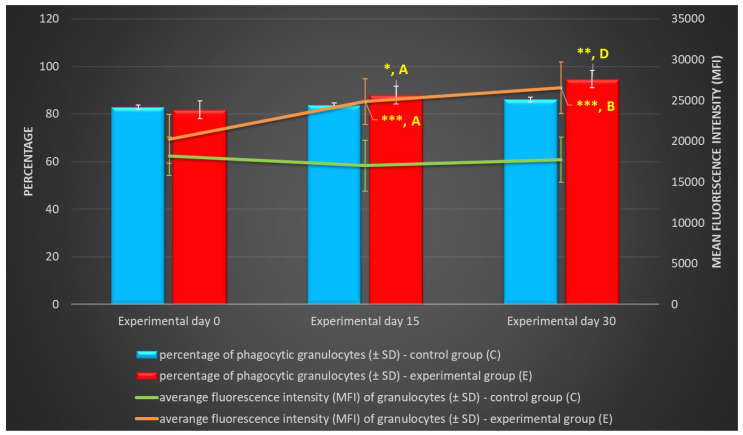
Percentage and mean fluorescence intensity (MFI) of phagocytic granulocytes in lamb groups in the Phagotest (mean ± SD). SD—standard deviation; numerical results are presented as the arithmetic mean ± SD. The significance levels were set at *p* ≤ 0.05 (*); *p* ≤ 0.01 (**); *p* ≤ 0.001 (***). Asterisk indicates strength of the significance between groups at given timepoints. Different letters between columns indicate strength of the difference within the group (A—*p* ≤ 0.05; B—*p* ≤ 0.01; D—*p* ≤ 0.0001) relative to day 0.

**Figure 2:**
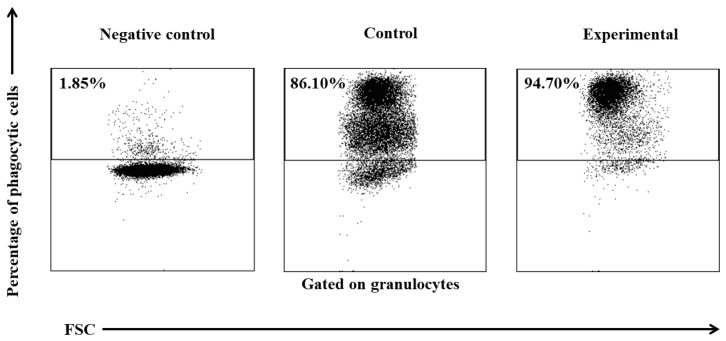
Dot plot cytogram showing the percentage of phagocytic granulocytes in control and experimental lambs on experimental day 30. Whole heparinized blood from control and experimental group animals was incubated for 10 min with FITC-labelled *E. coli* in an ice bath at a temperature of 0 °C (negative control) or in a water bath at a temperature of 37 °C (control and multi-strain probiotic). The percentages of granulocytes with ingested *E. coli* (FITC) bacteria were gated.

Based on the average phagocytic activity of peripheral blood neutrophil granulocytes in lambs, a significant increase (*p* ≤ 0.05 or *p* ≤ 0.01, respectively) in the average percentage of phagocytic neutrophils was noted on experimental days 15 and 30 (Table 1, Figure 1 and Figure 2) in the group of animals supplemented with the multi-strain probiotic relative to the control group not receiving any additives. The average percentage of phagocytic neutrophils in the peripheral blood of the lambs administered the probiotic also increased significantly on experimental days 15 and 30 (*p* ≤ 0.05 and *p* ≤ 0.0001, respectively) relative to day 0. Mean fluorescence intensity (MFI), a parameter denoting the number of bacteria ingested by one phagocyte, also increased significantly (*p* ≤ 0.001) between experimental days 15 and 30 (Table 1 and Figure 1) in the supplemented group relative to the control (non-supplemented) group. In the supplemented group, the MFI of peripheral blood neutrophils also increased significantly between experimental days 15 and 30 (*p* ≤ 0.05 or *p* ≤ 0.01, respectively) relative to day 0. 

### 2.2. Quantitative Determination of Monocytes Uptake of Bacteria (Phagocytosis) in Lambs

**Figure 3:**
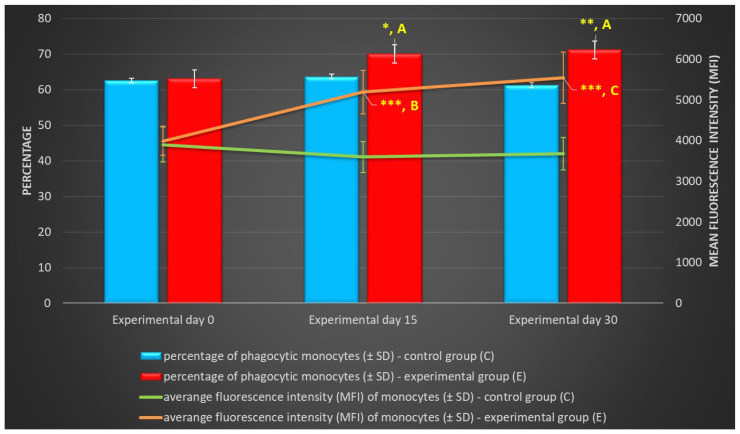
Percentage and mean fluorescence intensity (MFI) of phagocytic monocytes in lamb groups in the Phagotest (mean ± SD). SD—standard deviation; numerical results are presented as the arithmetic mean ± SD. The significance levels were set at *p* ≤ 0.05 (*); *p* ≤ 0.01 (**); *p* ≤ 0.001 (***). Asterisk indicates strength of the significance between groups at given timepoints. Different letters between columns indicate strength of the difference within the group (A—*p* ≤ 0.05; B—*p* ≤ 0.01; C—*p* ≤ 0.001) relative to day 0.

**Figure 4:**
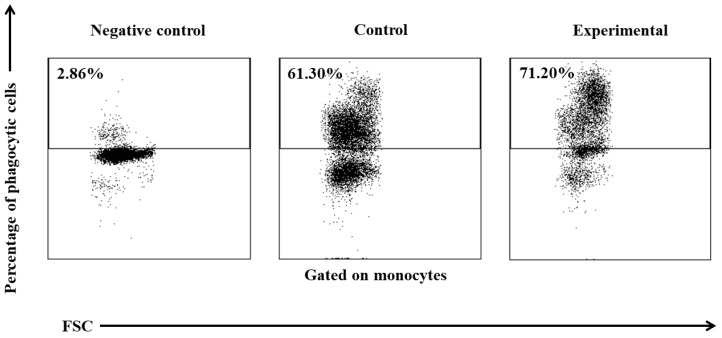
Dot plot cytogram showing the percentage of phagocytic monocytes in control and experimental lambs on experimental day 30. Whole heparinized blood from control and experimental group animals was incubated for 10 min with FITC-labelled *E. coli* in an ice bath at a temperature of 0 °C (negative control) or in a water bath at a temperature of 37 °C (control and multi-strain probiotic). The percentages of granulocytes with ingested *E. coli* (FITC) bacteria were gated.

Between experimental days 15 and 30, a similar but less significant increase (*p* ≤ 0.05 or *p* ≤ 0.01 or *p* ≤ 0.001) was also noted in the average percentage of phagocytic monocytes (Table 2, Figure 3 and Figure 4) and in the MFI (Figure 3) of peripheral blood monocytes in control and experimental lambs relative to the values noted on day 0. 

### 2.3. Quantitative Determination of Granulocytes’ Oxidative Burst in Lambs

**Figure 5:**
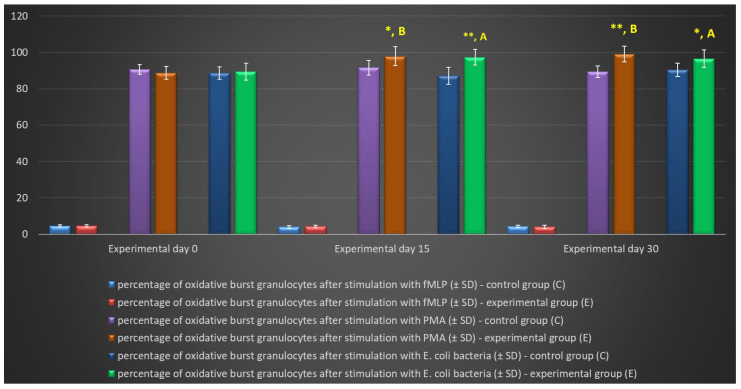
Percentage of granulocytes stimulated to undergo respiratory burst in lamb groups after stimulation with fMLP, PMA, and *E. coli* in the Phagoburst test (mean ± SD). SD—standard deviation; numerical results are presented as the arithmetic mean ± SD. The significance levels were set at *p* ≤ 0.05 (*); *p* ≤ 0.01 (**). Asterisk indicates strength of the significance between groups at given timepoints. Different letters between columns indicate strength of the difference within the group (A—*p* ≤ 0.05; B—*p* ≤ 0.01) relative to day 0.

**Figure 6:**
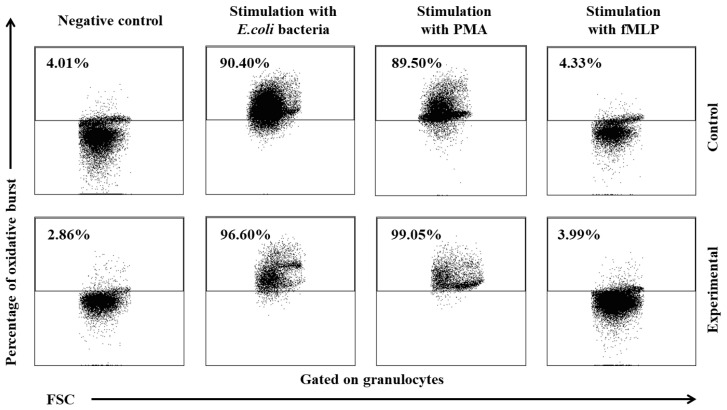
Dot plot cytogram showing the percentage of granulocytes stimulated to undergo respiratory burst in control and experimental lambs on experimental day 30. Whole heparinized blood from control group and experimental group (supplemented with the multi-strain probiotic) animals was divided into four test tubes. The samples were combined with the washing solution (negative control), *E. coli* bacteria (opsonizing stimulus), PMA (strong stimulus) or fMLP (weak stimulus), and incubated with dihydrorhodamine 123 in a water bath at a temperature of 37 °C. After incubation, cells were lysed and the DNA staining solution was added. The percentages of granulocytes stimulated to undergo respiratory burst (conversion of dihydrorhodamine 123 to rhodamine 123) were gated.

**Figure 7:**
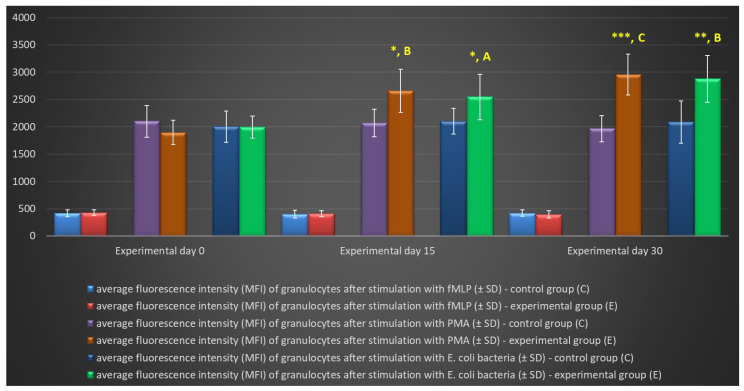
Mean fluorescence intensity (MFI) of granulocytes in lamb groups after stimulation with fMLP, PMA, and *E. coli* in the Phagoburst test (mean ± SD). SD—standard deviation; numerical results are presented as the arithmetic mean ± SD. The significance levels were set at *p* ≤ 0.05 (*); *p* ≤ 0.01 (**); *p* ≤ 0.001 (***). Asterisk indicates strength of the significance between groups at given timepoints. Asterisk indicates strength of the significance between groups at given timepoints. Different letters between columns indicate strength of the difference within the group (A—*p* ≤ 0.05; B—*p* ≤ 0.01; C—*p* ≤ 0.001) relative to day 0.

In the experimental group, the multi-strain probiotic exerted a positive influence on average respiratory burst activity (metabolism of highly reactive oxygen species-ROS) in peripheral blood neutrophil granulocytes relative to the control group and relative to the average values noted in the experimental group on day 0, throughout the experiment. An increase was observed in the average percentage of cells stimulated to undergo oxidative burst (Table 3, Figure 5 and Figure 6) as well as MFI describing the intensity of oxidative burst in various neutrophils (Figure 7). However, a significant increase (*p* ≤ 0.05 or *p* ≤ 0.01 or *p* ≤ 0.001) in the average values of the analysed parameters was noted between experimental days 15 and 30 only after simulation with strong oxidative burst activators (PMA and *E. coli* bacteria). In turn, no significant differences in these parameters were found during the experiment after stimulation with the weak oxidative burst activator (fMLP).

### 2.4. Quantitative Determination of Monocytes’ Oxidative Burst in Lambs

**Figure 8:**
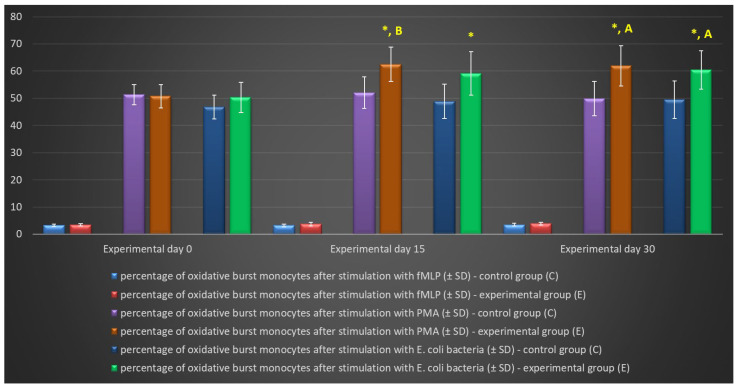
Percentage of monocytes stimulated to undergo respiratory burst in lamb groups after stimulation with fMLP, PMA, and *E. coli* in the Phagoburst test (mean ± SD). SD—standard deviation; numerical results are presented as the arithmetic mean ± SD. The significance levels were set at *p* ≤ 0.05 (*). Asterisk indicates strength of the significance between groups at given timepoint. Different letters between columns indicate strength of the difference within the group (A—*p* ≤ 0.05; B—*p* ≤ 0.01) relative to day 0.

**Figure 9:**
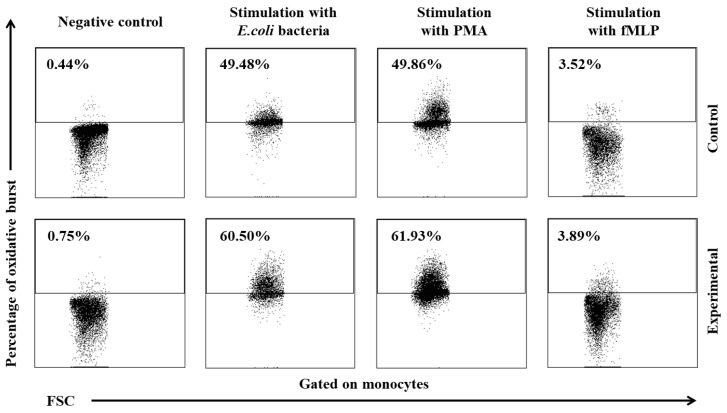
Dot plot cytogram showing the percentage of monocytes stimulated to undergo respiratory burst in control and experimental group lambs on experimental day 30. Whole heparinized blood from control group and experimental group (supplemented with the multi-strain probiotic) animals was divided into four test tubes. The samples were combined with the washing solution (negative control), *E. coli* bacteria (opsonizing stimulus), PMA (strong stimulus) or fMLP (weak stimulus), and incubated with dihydrorhodamine 123 in a water bath at a temperature of 37 °C. After incubation, cells were lysed and the DNA staining solution was added. The percentages of granulocytes stimulated to undergo respiratory burst (conversion of dihydrorhodamine 123 to rhodamine 123) were gated.

**Figure 10:**
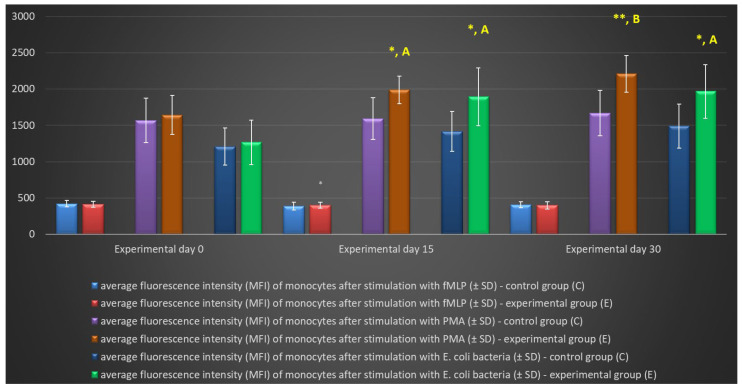
Mean fluorescence intensity (MFI) of monocytes in lamb groups after stimulation with fMLP, PMA, and *E. coli* in the Phagoburst test (mean ± SD). SD—standard deviation; numerical results are presented as the arithmetic mean ± SD. The significance levels were set at *p* ≤ 0.05 (*); *p* ≤ 0.01 (**). Asterisk indicates strength of the significance between groups at given timepoints. Different letters between columns indicate strength of the difference within the group (A—*p* ≤ 0.05; B—*p* ≤ 0.01) relative to day 0.

Similarly to granulocytes, the synthesis of highly ROS also increased when peripheral blood monocytes were stimulated to undergo oxidative burst with *E. coli*, PMA, and fMLP, and during the entire experiment, the average percentage of monocytes stimulated to undergo oxidative burst (Table 4, Figure 8 and Figure 9) and MFI (Figure 10) were significantly higher (*p* ≤ 0.05 or *p* ≤ 0.01) in the group of animals receiving the multi-strain probiotic than in the control (non-supplemented) group and in the experimental group relative to the values noted on day 0. Throughout the experiment, significant differences in the average percentage of monocytes capable of oxidative burst (Figure 8) and MFI in peripheral blood monocytes were not observed only after stimulation with the weak oxidative burst activator (fMLP) (Figure 10).

## 3. Discussion

The use of probiotics as feed additives in both monogastric and polygastric animals is not a new concept. For many years, researchers have been investigating the mechanism of action by which probiotics deliver health benefits and improve feed efficiency and performance in various animal species. These research efforts have been intensified in recent years, probably due to the continuous search for natural alternatives to antibiotics. In addition, research has shown that the efficacy of probiotics differs not only across microbial strains, but also across the supplemented macroorganisms. 

In healthy adults, the commensal gut microbiome consists of various microorganisms that colonize a specific segment of the gastrointestinal tract [29,30]. These microorganisms can survive under changing environmental conditions because they strongly adhere to the intestinal epithelium and cannot be easily eliminated from the body [31]. These microorganisms not only strengthen local immune defences against infection (GALT), but also modulate the systemic immune response. Microbe-specific molecules, including microbe-associated molecular patterns (MAMPs), their metabolites, and other signalling molecules, are released in the intestines and transferred from the intestinal lumen to the circulatory system. These molecules stimulate immunocompetent cells and prepare them for potential pathogen invasion [30,32]. However, the gut microbiome undergoes numerous changes, in particular in newborns, under the influence of both external and internal factors. In suckling animals, the digestive system is not yet fully colonized by healthy microbiota, and the immune system is not yet fully developed, which makes them more susceptible to infections than adult organisms [33]. Weaning and the transition from mother’s milk to concentrate feed is one of the critical moments in the early life of mammals, including lambs. Weaning often induces changes in immune system function, which can increase the prevalence of disease and decrease performance in lambs. The negative effects of weaning can be minimized by supplementing lamb diets with various probiotic strains. Dietary supplementation usually promotes gut colonization by commensal strains that increase intestinal barrier integrity, alleviate inflammations, and decrease the number of foreign particles in circulating blood, such as bacteria, viruses, dead cells, and other undesirable substances [32]. 

Phagocytosis, a key mechanism of cellular innate immunity, is one of the processes that is responsible for removing these antigens from the body. Phagocytosis involves phagocytes that are able to identify and eliminate foreign and infected cells. These cells include both professional phagocytes (polymorphonuclear neutrophils, monocytes, monocyte-derived macrophages, and tissue-resident macrophages) and non-professional phagocytes (epithelial cells, fibroblasts, and dendritic cells). Professional phagocytes are rapidly mobilized and accumulate in large numbers in the site of infection. In turn, non-professional phagocytes are less effective, have a limited range of target particles, and eliminate antigens less rapidly than professional phagocytes [4,34]. Phagocytosis is a multi-stage process that is initiated by pattern recognition receptors (PRRs), including dectin-1, Toll-like receptor 2 (TLR-2), complement receptor 3 (CRS3), lactosylceramide (CDw17), class A scavenger receptors (SRs), SR-A/CD204, macrophage receptor with collagenous structure (MARCO), and probably other receptors that can identify and bind specific pathogen-associated molecular patterns (PAMPs) or microorganisms [35,36]. A phagocyte engulfs the targeted molecule and forms a phagosome which then fuses with a lysosome to create a phagolysosome. The following intracellular killing mechanisms are initiated: non-oxidative mechanisms (not dependent on oxygen), such as the activation of basic proteins and hydrolytic enzymes, and oxidative (oxidative burst) mechanisms that produce ROS. During digestion, enzymes decompose foreign particles into simple chemical compounds that are removed from the phagocyte. In some cases, especially when phagocytes engulf bacteria and viruses, cells present fragments of foreign proteins (epitopes) on their surface. As a result, other immune cells can identify foreign particles and initiate the immune response [37]. 

The above explains the significant increase in the phagocytic activity of monocytes and granulocytes, expressed by the percentage of phagocytic cells and the average number of bacteria eliminated by one phagocyte (MFI), in the group of lambs supplemented with the multi-strain probiotic formulation relative to the control group. This observation suggests that probiotic strains not only increase the number of phagocytic cells, but also enhance the effectiveness of phagocytosis. Phagocytes also play an important role in triggering and regulating the specific immune response via cytokines and reactive intermediates released by macrophages [38]. A review of the literature indicates that probiotics exert a highly selective and strain-specific effect on cellular immunity, and that phagocytosis is stimulated by only a limited number of strains. An increase in the activity of phagocytic cells was reported by Devyatkin et al. [39] in sheep and lambs whose diets were supplemented with spore-forming *Bacillus subtilis* and *Bacillus licheniformis* bacteria for 30 days. Similar observations were made by Kausahal et al. [40] in a study of mice whose diets were supplemented with *Lactobacillus acidophilus* and *Bifidobacterium bifidum,* and by Arunachalam et al. [41] in a study of humans who consumed milk containing *Bifidobacterium lactis* (HN019) for 6 weeks. In the former and latter study, old age did not affect the phagocytic potential of macrophages and polymorphonuclear cells, respectively. Rocha-Ramírez et al. [42] examined the effect of four probiotic *Lactobacillus* strains (*L. rhamnosus* GG, *L. rhamnosus* KLSD, *L. helveticus* IMAU70129, and *L. casei* IMAU60214) on the activity of human macrophages in vitro. They found that the phagocytic activity of macrophages targeting both extracellular (*Staphylococcus aureus* and *Escherichia coli*) and intracellular pathogens (*Salmonella typhimurium*) was intensified after 1 h of preincubation with heat-inactivated probiotic strains at a temperature of 37 °C. In an ex vivo study, Gill et al. [43] also observed an increase in the phagocytic activity of mononuclear and polymorphonuclear cells in humans after the consumption of *Bifidobacterium lactis* HN019, in particular in immunocompromised individuals. In turn, Ren et al. [44] conducted an in vivo study of mice supplemented with *Lactobacillus salivarius* CICC 23,174 or *Lactobacillus plantarum* CGMCC 1.557 bacterial strains for 20 days and reported a highly significant, nearly 30% increase in the phagocytosis index (PI), but only in mice receiving *L. plantarum* at a dose of 1 × 10^9^ CFU/animal relative to the control group. 

In the current study, the multi-strain probiotic formulation also intensified the intracellular killing activity of granulocytes and monocytes stimulated with PMA and *E. coli*, expressed as the percentage of stimulated cells and MFI. Similar observations were made by Kapila et al. [45] in mice that were fed milk fermented with *Lactobacillus helveticus* NCDC292, *L. acidophilus* NCDC15, or *L. paracasei* for 60 days. In the cited study, the activity of neutrophil respiratory burst enzymes (cytochrome C reductase and myeloperoxidase), the activity of β-galactosidase and β-glucuronidase enzymes, and nitric oxide production increased during the first 30 days of the experiment, which enhanced the phagocytic activity of neutrophils and macrophages. As previously mentioned, Rocha-Ramirez et al. [42] found that the challenge with lactic acid bacteria was not only the increase in the phagocytic activity of macrophages, but also the stimulation of mononuclear phagocytes (monocytes and macrophages) to produce ROS, depending on the concentration and species of bacteria. The present findings are also corroborated by the results of a study conducted on mouse macrophages [46] which released large amounts of ROS after stimulation with three *Lactobacillus* strains (*L. reuteri* 115, *L. johnsonii* 142, ad *L. animalis/murinus* 148). In turn, a study examining the effect of macrophage stimulation with three *Lactobacillus paracasei* strains (KW3110, ATCC53103, and NRIC1942) [47] revealed a clear correlation between phagocytosis and the production of ROS and, consequently, IL-12. Another study demonstrated that *Lactobacillus rhamnosus* GG and *Lactobacillus paracasei* Fn032 strains exerted antioxidant effects and modulated the redox status of a colonic fermentation system, which is related to their radical scavenging ability or antibacterial effects [48].

According to Donnet-Hughes et al. [49], the ambiguous effect of various probiotic strains on phagocytosis, a mechanism of cellular innate immunity, could be directly linked with the fact that these cells are activated by surface receptors, and it could be indirectly associated with the release of cytokines from immunocompetent cells. Despite differences in the terminology associated with PAMPs and MAMPs, these structures (lipopolysaccharide, LPS, peptidoglycan, and lipoteichoic acids) are often similar, and they are equally identified and bound by nucleotide binding and oligomerization domain-containing protein 2 (NOD2)-like receptors (NLRs) and Toll-like receptors (TLRs). These receptors belong to the group of PRRs that are characteristic of innate immune cells, including phagocytic cells [50]. However, the mechanism by which the immune system differentiates healthy microbiota from pathogenic bacteria remains unknown. According to Gaboriau-Routhiau et al. [51], and Manicassamy et al. [52], functional TLRs are also present in T cells, and direct triggering on TLRs can occur. There is considerable evidence to indicate that some probiotic bacteria can stimulate the anti-inflammatory pattern of cytokines (IL-10, TGF-β) via TLRs, whereas other probiotic strains, similarly to pathogenic bacteria, induce the production of proinflammatory cytokines (TNF-α, IL-12p70, and IL-23) that are responsible for the inflammatory response. A prolonged or excessive inflammatory response can damage tissues around the infection site and lead to defects in the intestinal barrier [53,54,55,56,57,58,59,60]. The cells of the innate immune system, including phagocytes, produce and release cytokines and other factors that stimulate the differentiation of naïve T cells into subpopulations of effector Th1, Th2 Th17, or regulatory (T_reg_) lymphocytes (TR1 and Th3) [33]. Researchers generally agree that most TLR signalling pathways strongly stimulate macrophages and dendritic cells to produce IL-12 (p70), which promotes the differentiation of Th0 cells to Th1 cells and the production of interferon (IFN-γ) by Th1 cells [61,62]. Interferon is essential in the initial phase of a bacterial infection because it promotes the phagocyte-dependent protective response and suppresses a Th2-type humoral response [63,64]. IL-12 is an important cytokine that induces a Th1-type immune response and increases cellular immunity [65], but according to Bafica et al. [66] and Ichikawa et al. [67], this correlation can be ambiguous. Velez et al. [68] and Hua et al. [69] demonstrated that various probiotic strains are able to stimulate a Th1-type immune response. Th1 cells promote the activation of phagocytes and antimicrobial training. In addition, TLR1, TLR2, and TLR4 bind the structures of probiotic bacteria and can also influence ROS production in macrophages [70,71]. Pelto et al. [72] have suggested that an increase in the expression of complement receptors that play an important role in phagocytosis, including CR1, CR3, FcγRII, and FcαR, on neutrophils can also intensify the phagocytic activity of peripheral blood mononuclear cells (PBMCs). Similarly to bacterial LPS and viral products, proinflammatory cytokines acting via transcriptional factors NF-κB and AP-1 can also stimulate the secretion of IL-8 (CXCL-8), a chemokine that is responsible for the migration of phagocytes (neutrophils and macrophages) to the site of inflammation [73].

Despite extensive research into probiotics’ immunomodulatory effects on immune system functions, including the phagocytic activity of neutrophils and monocytes, many processes remain unknown and require further study. It should be noted that probiotics can exert different effects on phagocytosis, depending on the applied strain, the administered dose, and individual factors. Not all probiotics exert identical immunomodulatory effects, and further research is needed to elucidate their specific mechanisms of action. 

## 4. Materials and Methods

### 4.1. Experimental Design

This study was conducted on a flock of Kamieniec sheep in a farm in Komalwy in the Region of Warmia and Mazury, Poland. Sixteen young rams, the offspring of 3-year-old ewes, were selected for the experiment. Lambs were divided into two groups of 8 animals each, a control group (group C) and an experimental group (group E), by the analogue method, based on their body weights at 10 days of age. The two groups were similar in body weights. The animals from both groups were slaughtered at the end of the experiment, i.e., at 40 days of age. Lambs were kept in two pens. During the experiment, the animals could move freely and had access to water free of antibiotics.

In both groups, the ewes and the lambs were fed identical diets according to the feeding system adopted in the farm. The ewes had ad libitum access to a total mixed ration (TMR) composed of grass silage (64%), maize silage (32%), concentrate (3.5%), and Milafos L mineral and vitamin premix (0.5%). Concentrate feed was prepared from ground oats (50%), ground wheat (30%), ground maize (10%), and ground soybeans (10%). The animals had unlimited access to Multi-Lisal Se mineral licks. Lambs were naturally fed colostrum in the first hour of life, and their diets in the first 10 days of life consisted solely of the mother’s milk. Lambs were provided with ad libitum access to concentrate feed at 11 days of age. Group C lambs received a basal diet, whereas group E lambs received a basal diet supplemented with probiotics as described in the details below. 

Beginning at 11 days of age, the diets of group E lambs were supplemented with a multi-strain probiotic formulation. The probiotic formulation contained four bacterial strains, including three *Lactobacillus* stains (*Lactobacillus plantarum* AMT14, *Lactobacillus plantarum* AMT4, and *Lactobacillus rhamnosus* AMT15) and one *Bifidobacterium animalis* AMT30 strain (Nature Science, Stawiguda, Poland) with a viable count of 1.0 × 10^9^ CFU/g. An aqueous solution of the probiotic formulation was administered per os once daily to each lamb according to the following schedule: age of 11–20 days—10 mL of the solution (1 g of the multi-strain probiotic/animal), age of 21–30 days—10 mL of the solution (2 g of the multi-strain probiotic/animal), and age of 31–40 days—10 mL of the solution (3 g of the multi-strain probiotic/animal).

### 4.2. Sample Collection

Blood for analyses was sampled from the jugular vein (around 10 mL of fresh blood) at the beginning of the experiment before supplementation with the multi-strain probiotic (day 0), and on days 15 and 30 of the experiment. Blood samples were used to determine the phagocytic activity (Phagotest^®^ kit) (Glycotope Biotechnology GmbH, Heidelberg, Germany) and oxidative metabolism (Phagoburst^®^ kit) (Glycotope Biotechnology GmbH, Heidelberg, Germany) of peripheral blood granulocytes and monocytes by flow cytometry. All blood samples were collected before the morning feeding. 

### 4.3. Determination of the Phagocytic Activity of Blood Granulocytes and Monocytes in Lambs with the Phagotest^®^ kit

All test reagents were prepared in accordance with the manufacturer’s recommendations in the leaflet attached to the product. One hundred microlitres of whole heparinized blood chilled to 0 °C and 20 μL of chilled *E. coli* bacteria (Glycotope Biotechnology GmbH, Heidelberg, Germany) were added to each of the two 5 mL test tubes (blue, Beckman Coulter, Fullerton, CA, USA) (negative control and experimental samples) and shaken for around 3 s at low speed. The experimental sample was incubated for 10 min at 37 °C, and the negative control sample was incubated in an ice bath at 0 °C. After incubation, 100 μL of a quenching solution (Glycotope Biotechnology GmbH, Heidelberg, Germany) was added to each sample, and the samples were shaken. Three mL of the washing solution (Glycotope Biotechnology GmbH, Heidelberg, Germany) chilled to 0 °C was added; the samples were centrifuged for 5 min at 4 °C (250× *g*), and the supernatant was removed. The rinsing procedure was performed twice, and 2 mL of the lysing solution (Glycotope Biotechnology GmbH, Heidelberg, Germany) at room temperature was added to each sample. The samples were shaken and incubated at room temperature for 20 min. The samples were centrifuged for 5 min at 4 °C (250× *g*), and the supernatant was removed. Three millilitres of the washing solution (Glycotope Biotechnology GmbH, Heidelberg, Germany) chilled to 0 °C was added to each sample; the samples were centrifuged for 5 min at 4 °C (250× *g*), and the supernatant was removed. Two hundred microlitres of the DNA staining solution (Glycotope Biotechnology GmbH, Heidelberg, Germany) chilled to 0 °C was added; the samples were shaken and incubated for 10 min in an ice bath. Cellular phagocytic activity was determined in a cytometer (FACSCelesta cytometer, BD Biosciences, San Jose, NJ, USA) in less than 60 min after the last reagent had been added. The Phagotest (Glycotope Biotechnology GmbH, Heidelberg, Germany) involved fluorescein (FITC)-stained *E. coli* bacteria, which are phagocytized by macrophages. Cell nuclei were also stained. The test determines the number of phagocytizing cells, granulocytes, and monocytes separately, and their phagocytic activity, i.e., the number of bacteria ingested by a single cell, which is expressed by MFI.

### 4.4. Determination of the Oxidative Metabolism of Blood Granulocytes and Monocytes in Lambs with the Phagoburst^®^ Kit

All test reagents were prepared in accordance with the manufacturer’s recommendations in the leaflet attached to the product. Each analysed sample of whole heparinized blood was divided into four test tubes (blue, Beckman Coulter, Fullerton, CA, USA) of 100 μL each and chilled to 0 °C. A total volume of 20 μL of chilled *E. coli* bacteria (Glycotope Biotechnology GmbH, Heidelberg, Germany) was added to the first sample (experimental), 20 μL of the washing solution (Orpegen Pharma, Heidelberg, Germany) was added to the second sample (negative control), 20 μL of fMLP (N-formyl-methionyl-leucyl-phenylalanine) (Glycotope Biotechnology GmbH, Heidelberg, Germany) was added to the third sample (low control), and 20 μL of PMA (4-phorbol-12-β-myristate-13-acetate) (Glycotope Biotechnology GmbH, Heidelberg, Germany) was added to the fourth sample (high control). Test tube contents were stirred and incubated for 10 min at 37 °C (excluding the fMLP (Glycotope Biotechnology GmbH, Heidelberg, Germany) sample which was incubated for 7 min). After incubation, each test tube was supplemented with 20 μL of the substrate solution (Glycotope Biotechnology GmbH, Heidelberg, Germany) and thoroughly shaken. All samples were incubated for 10 min at 37 °C. After incubation, 2 mL of the lysing solution (Glycotope Biotechnology GmbH, Heidelberg, Germany) with room temperature was added. The test tubes were shaken and incubated at room temperature for 20 min. All samples were centrifuged for 5 min at 4 °C (250× *g*), and the supernatant was removed. All test tubes were rinsed once with 3 mL of the washing solution (Glycotope Biotechnology GmbH, Heidelberg, Germany), centrifuged for 5 min at 4 °C (250× *g*), after which the supernatant was removed. Two hundred μL of the staining solution chilled to 0 °C was added to each sample; the test tubes were shaken and incubated for 10 min in an ice bath. The intracellular killing activity of phagocytes was determined in a cytometer (FACSCelesta cytometer, BD Biosciences, San Jose, NJ, USA) in less than 30 min after the last reagent had been added. Three activators were used to stimulate cells: *E. coli* bacteria (Glycotope Biotechnology GmbH, Heidelberg, Germany), PMA (Glycotope Biotechnology GmbH, Heidelberg, Germany) as the strong activator, and fMLP (Glycotope Biotechnology GmbH, Heidelberg, Germany) as the weak activator. Dihydrorodamine (123-DHR) was oxidized by mitochondria when H_2_O_2_ was added to induce oxidative stress, and it was converted to cation rhodamine 123 (R123), the fluorescence emitter.

### 4.5. FACS Acquisition and Analysis

**Figure 11:**
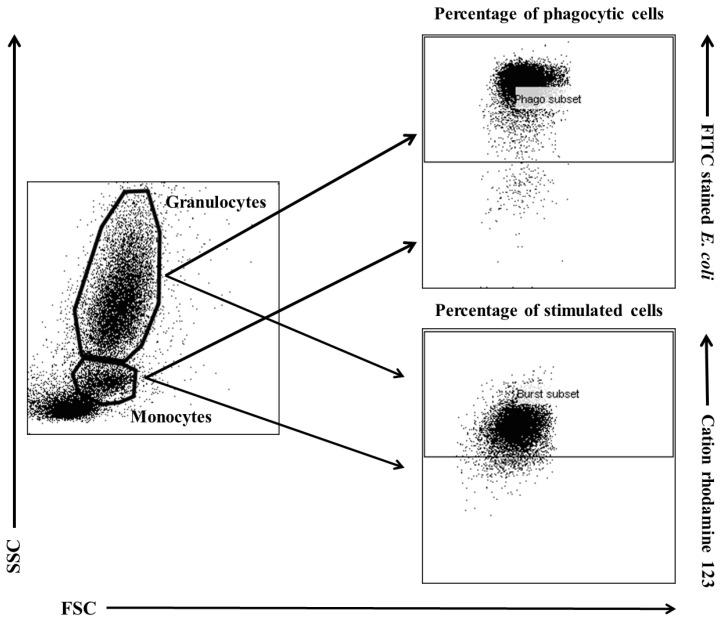
Gating strategy for analysing flow cytometry data. Granulocytes and monocytes were gated based on forward and side scatter (FSC/SSC) parameters. Each cell subset was analysed for the relative number of phagocytizing cells and cells stimulated for respiratory burst (fMLP, PMA, or *E. coli* bacteria).

Flow cytometry was performed with a FACSCelesta cytometer (BD Biosciences, San Jose, NJ, USA). Data were acquired with FACSDiva version 6.1.3 software (BD Biosciences, San Jose, NJ, USA) and analysed in FlowJo 10 software (Tree Star, Ashland, OR, USA). The cytometry setup and tracking beads (CST; BD Biosciences, San Jose, NJ, USA) were used to initialize the photomultiplier tube (PMT). Unstained control cells and a single stain control for every fluorochrome were prepared and used to establish flow cytometric compensation (Figure 11).

### 4.6. Statistical Analysis

Data were systematically compiled into Excel spreadsheets for subsequent statistical analysis. To validate the suitability of parametric statistical methods for our analysis, we first assessed the normality of distributions and the homogeneity of variances across all study groups. This verification was accomplished using the Shapiro–Wilk test for normality and the Levene test for homogeneity of variances, where a *p*-value indicative of non-significant deviation from normality or homogeneity affirmed the use of parametric tests. Following confirmation of these assumptions, parametric analyses were employed. The numerical data were expressed as arithmetic means ± standard deviation (SD). For inferential statistics, a two-way ANOVA for orthogonal designs was applied to identify any significant differences within the experimental framework. Post hoc comparisons were conducted using Dunnett’s test to assess changes over time within group E, com-paring baseline (day 0) against subsequent measurements on days 15 and 30. Statistical significance was denoted as follows: (A) *p* < 0.05; (B) *p* < 0.01; (C) *p* < 0.001; (D) *p* < 0.0001. Additionally, Tukey’s test was used for intergroup comparisons between group E and control group C at each time interval, with results detailed to three decimal places in the tables. All statistical computations were performed using GraphPad Prism version 7 software.

## 5. Conclusions

In recent years, the demand for feed additives that not only promote optimal growth, but also boost immunity in animals has increased in livestock production, including in sheep farms. Probiotics may not be novel feed additives, but they meet the above criteria. Over the years, various types of probiotics have been studied in different animal species, and the results are inconclusive because the efficacy of probiotics depends on the applied microorganism and the supplemented macroorganisms. 

In the present study, a significant increase in phagocytic activity (expressed as the percentage of phagocytes), average number of bacteria eliminated by one phagocyte (expressed as the mean fluorescence intensity of monocytes and granulocytes), and intracellular killing activity of granulocytes and monocytes stimulated with PMA and *E. coli* (expressed as the percentage of stimulated cells and mean fluorescence intensity) was observed in the group of experimental lambs whose diets were supplemented with a multi-strain probiotic formulation (*Lactobacillus plantarum* AMT14, *Lactobacillus plantarum* AMT4, *Lactobacillus rhamnosus* AMT15, and *Bifidobacterium animalis* AMT30) relative to control group animals without feed supplementation. These findings suggest that the tested probiotic formulation may have a positive effect on the immune status of lambs, although further and more complex investigation is required. 

## Figures and Tables

**Table 1:** Percentage of phagocytic granulocytes and mean fluorescence intensity of granulocytes in lamb groups in the Phagotest (mean ± SD).

Granulocytes	Day	Group		
		Control (C)	Experimental (E) ^1^	*p*-Value
% Phagocytic cells	0	82.90 ± 3.23	81.80 ± 4.09	0.560
	15	83.80 ± 3.71	87.90 ± 2.53 ^A^	0.022
	30	86.10 ± 4.14	94.70 ± 3.03 ^D^	*p* < 0.001
Mean fluorescence intensity	0	18,167.33 ± 2364.66	20,261.67 ± 2997.65	0.143
	15	16,998.83 ± 3124.37	24,880.17 ± 2806.23 ^A^	*p* < 0.001
	30	17,723.67 ± 2767.42	26,549.67 ± 3161.47 ^B^	*p* < 0.001

SD–standard deviation; numerical results are presented as the arithmetic mean ± SD. ^1^ Different letters within columns indicate strength of the difference within the group (^A^—*p* ≤ 0.05; ^B^—*p* ≤ 0.01; ^D^—*p* ≤ 0.0001) relative to day 0.

**Table 2:** Percentage of phagocytic monocytes and the mean fluorescence intensity of monocytes in lamb groups in the Phagotest (mean ± SD).

Monocytes	Day	Group		
		Control (C)	Experimental (E) ^1^	*p*-Value
% Phagocytic cells	0	62.55 ± 3.24	63.10 ± 3.02	0.730
	15	63.70 ± 4.26	70.05 ± 3.97 ^A^	0.008
	30	61.30 ± 4.84	71.20 ± 5.58 ^A^	0.002
Mean fluorescence intensity	0	3899.17 ± 428.38	3989.67 ± 351.14	0.651
	15	3592.67 ± 377.51	5192.67 ± 536.87 ^B^	*p* < 0.001
	30	3676.26 ± 394.41	5544.33 ± 629.66 ^C^	*p* < 0.001

SD—standard deviation; numerical results are presented as the arithmetic mean ± SD. ^1^ Different letters within columns indicate strength of the difference within the group (^A^—*p* ≤ 0.05; ^B^—*p* ≤ 0.01; ^C^—*p* ≤ 0.001) relative to day 0.

**Table 3:** Average intracellular killing activity of granulocytes and mean fluorescence intensity in lamb groups after stimulation with fMLP, PMA, and *E. coli*, as determined in the Phagoburst test (mean ± SD).

Granulocytes		Day	Group		
			Control (C)	Experimental (E) ^1^	*p*-Value
fMLP	% Stimulated cells	0	4.51 ± 0.67	4.64 ± 0.65	0.697
		15	3.94 ± 0.83	4.26 ± 0.59	0.390
		30	4.33 ± 0.71	3.99 ± 0.87	0,406
	Mean fluorescence intensity	0	415.33 ± 62.31	425.83 ± 49.57	0.716
		15	395.89 ± 71.38	406.07 ± 52.47	0.750
		30	420.05 ± 58.03	394.51 ± 68.17	0.434
PMA	% Stimulated cells	0	90.63 ± 2.73	88.78 ± 3.47	0.256
		15	91.57 ± 4.03	97.95 ± 5.13 ^B^	0.015
		30	89.50 ± 3.17	99.05 ± 4.28 ^B^	*p* < 0.001
	Mean fluorescence intensity	0	2101.23 ± 288.53	1,897.38 ± 221.36	0.135
		15	2069.77 ± 254.19	2,661.33 ± 394.17 ^B^	0.003
		30	1968.55 ± 239.41	2,957.62 ± 377.35 ^C^	*p* < 0.001
*E. coli* bacteria	% Stimulated cells	0	88.60 ± 3.47	89.49 ± 4.65	0.671
		15	87.22 ± 4.66	97.40 ± 4.32 ^A^	*p* < 0.001
		30	90.40 ± 3.76	96.60 ± 4.83 ^A^	0.012
	Mean fluorescence intensity	0	2004.17 ± 287.53	1994.33 ± 198.68	0.939
		15	2103.64 ± 237.81	2547.00 ± 415.45 ^A^	0.020
		30	2089.27 ± 387.61	2874.67 ± 429.72 ^B^	0.002

SD—standard deviation; numerical results are presented as the arithmetic mean ± SD. ^1^ Different letters within columns indicate strength of the difference within the group (^A^—*p* ≤ 0.05; ^B^—*p* ≤ 0.01; ^C^—*p* ≤ 0.001) relative to day 0.

**Table 4:** Average intracellular killing activity of monocytes and mean fluorescence intensity in lamb groups after stimulation with fMLP, PMA, and *E. coli* in the Phagoburst test (mean ± SD).

Monocytes		Day	Group		
			Control (C)	Experimental (E) ^1^	*p*-Value
fMLP	% Stimulated cells	0	3.25 ± 0.37	3.43 ± 0.41	0.372
		15	3.21 ± 0.52	3.71 ± 0.67	0.118
		30	3.52 ± 0.49	3.89 ± 0.46	0.142
	Mean fluorescence intensity	0	422.64 ± 42.21	413.53 ± 38.23	0.658
		15	388.51 ± 53.02	402.67 ± 41.02	0.560
		30	406.67 ± 39.89	397.95 ± 50.03	0.706
PMA	% Stimulated cells	0	51.37 ± 3.71	50.78 ± 4.27	0.772
		15	52.12 ± 5.85	62.52 ± 6.35 ^B^	0.004
		30	49.86 ± 6.33	61.93 ± 7.45 ^A^	0.004
	Mean fluorescence intensity	0	1567.23 ± 305.43	1642.03 ± 267.64	0.611
		15	1591.78 ± 286.34	1989.69 ± 188.24 ^A^	0.005
		30	1668.53 ± 311.06	2209.36 ± 252.55 ^B^	0.002
*E. coli* bacteria	% Stimulated cells	0	46.83 ± 4.37	50.27 ± 5.55	0.191
		15	48.90 ± 6.35	59.10 ± 7.98	0.013
		30	49.48 ± 6.88	60.50 ± 7.07 ^A^	0.007
	Mean fluorescence intensity	0	1209.00 ± 253.21	1266.05 ± 306.47	0.691
		15	1415.33 ± 273.25	1894.67 ± 397.32 ^A^	0.014
		30	1489.50 ± 302.33	1968.34 ± 368.51 ^A^	0.013

SD—standard deviation; numerical results are presented as the arithmetic mean ± SD. ^1^ Different letters within columns indicate strength of the difference within the group (A—*p* ≤ 0.05; B—*p* ≤ 0.01), relative to day 0.

## Data Availability

All relevant data are contained within the manuscript.
